# Prokaryotic soluble expression and purification of bioactive human fibroblast growth factor 21 using maltose-binding protein

**DOI:** 10.1038/s41598-017-16167-x

**Published:** 2017-11-23

**Authors:** Anh Ngoc Nguyen, Jung-A Song, Minh Tan Nguyen, Bich Hang Do, Grace G. Kwon, Sang Su Park, Jiwon Yoo, Jaepyeong Jang, Jonghwa Jin, Mark J. Osborn, Yeon Jin Jang, Thu Trang Thi Vu, Heung-Bum Oh, Han Choe

**Affiliations:** 1 Department of Physiology, Asan-Minnesota Institute for Innovating Transplantation, Bio-Medical Institute of Technology, University of Ulsan College of Medicine, Asan Medical Center, Seoul, 05505 Korea; 2 New Drug Development Center, Osong Medical Innovation Foundation, 123 Osong Saengmyung-Ro, Cheongju, Chungcheongbuk-Do 28160 Korea; 3 Department of Pediatrics, Division of Blood and Marrow Transplantation, Center for Genome Engineering, Stem Cell Institute, University of Minnesota, Minneapolis, MN 55455 USA; 4 Department of Physiology, Cell Dysfunction Research Center, University of Ulsan College of Medicine, Seoul, 05505 Korea; 5 Department of Laboratory Medicine, University of Ulsan College of Medicine and Asan Medical Center, Seoul, 05505 Korea

## Abstract

Human fibroblast growth factor 21 (hFGF21) has been characterized as an important regulator of glucose and lipid metabolism homeostasis. Here, to produce hFGF21 efficiently in *Escherichia coli*, the expression and solubility of hFGF21 were tested and optimised by fusing the protein with one of eight tags: hexahistidine (His6), thioredoxin (Trx), small ubiquitin-related modifier (Sumo), glutathione S-transferase (GST), maltose-binding protein (MBP), N-utilisation substance protein A (NusA), human protein disulphide isomerase (PDI), and the b′a′ domain of PDI (PDIb′a′). Each tag increased solubility of the protein when the expression temperature was 18°C. Unlike many other tags that were tested, MBP significantly enhanced the solubility of the protein also in the culture condition at 37°C. Thus, the MBP-hFGF21 construct was further pursued for optimisation of affinity chromatography purification. After tag removal, 8.1 mg of pure hFGF21 was obtained as a final product from 500 mL of starting culture. The protein was then characterised by mass spectroscopy and an *in vitro* functional assay using NIH-3T3 cells transfected with a β-klotho reporter gene. These characteristics are similar to those of commercial hFGF21. Thus, the MBP tag is useful for efficient prokaryotic production and purification of bioactive hFGF21.

## Introduction

Fibroblast growth factor 21 (FGF21) is a member of the FGF family and contains a 28-amino acid signal peptide (181 amino acid residues total as the mature FGF21 peptide)^1,2^. It is synthesised by multiple organs such as the liver, heart, brain, adipose, and pancreas^2^. The protein stimulates glucose incorporation and has been shown to dramatically lower fasting glucose, insulin, and plasma fructosamine concentrations in diabetic rhesus monkeys^3–5^. Alternate isoforms of FGF21 show the ability to reduce weight and lower serum lipid levels in patients with obesity and type 2 diabetes^6^. Furthermore, it has anti-inflammatory functions in the cardiac muscle and pancreas^2^. Given the diverse roles of FGF21, it is a promising therapeutic agent for the treatment of metabolic syndrome^5,7,8^. Previous attempts to produce human FGF21 (hFGF21) have mandated high cost approaches that nevertheless result in suboptimal recovery of bioactive protein^3,9,10^.

*Escherichia coli* is the most popular expression host because it can grow rapidly to high cell densities in inexpensive media and under many circumstances results in a high production yield of a recombinant protein^11^. Nonetheless, the high expression of some recombinant proteins in *E. coli* often leads to misfolded and aggregated proteins called inclusion bodies^11,12^. This scenario has limited the production of hFGF21 in *E. coli* because of formation of the non-functional inclusion bodies, which require time-consuming optimisation for solubilisation, refolding, and purification to obtain the active peptide^3,10,13^. Previous efforts to overcome such drawbacks have employed a small ubiquitin-related modifier (Sumo) that was fused with hFGF21 to facilitate the soluble expression and purification of the protein^10,14^. In another study, hFGF21 was expressed in soluble form under optimised fermentation conditions; however, the protein was purified without removal of its hexahistidine tag^15^. Eukaryotic production platforms allow for soluble expression of hFGF21^9,16^, however, the downside is poor productivity, protein hyper-glycosylation, and proteolysis at the N terminus of the peptide^16^. For optimizing the efficient production of the native hFGF21 protein, here we studied the ability of tag sequences to enhance the expression and solubility of FGF21 in *E. coli*. Eight tag genes were utilised: hexahistidine (His6), small ubiquitin-related modifier (Sumo), thioredoxin (Trx), glutathione S-transferase (GST), maltose-binding protein (MBP), N-utilisation substance protein A (NusA), human protein disulphide isomerase (PDI), and the b′a′ domain of PDI (PDIb′a′). Each tag candidate was attached to the N terminus of hFGF21 and among these, the MBP tag significantly improved protein expression and solubility. Finally, to obtain bioactive hFGF21, the MBP-hFGF21 fusion was purified by a single conventional chromatography technique followed by tag cleavage. This method resulted in a maximal yield of 8.1 mg of hFGF21 from 500 mL of cell culture in highly pure form with negligible endotoxin levels. Moreover, the FGF21 purified under these novel conditions increased the proliferation of NIH-3T3 cells transfected with β-klotho thus proving functionality of the recombinant protein.

## Materials and Methods

### Materials

Coomassie brilliant blue R-250, and Tris base were purchased from Amresco (Solon, Ohio). 1-Thio-β-d-galactopyranoside (IPTG) was acquired from Anaspec (Fremont, CA), and imidazole from Deajung Chemicals (Siheung, Korea). Ampicillin was acquired from Duchefa Biochemie (Haarlem, Netherlands), and NaCl and glycerol from Samchun Chemical (Pyongtaek, Korea). The HisTrap FF, HiLoad 26/60 Superdex 75 PG columns, and HiPrep 16/10 Desalting column were purchased from GE healthcare (Piscataway, NJ). A protein-pak 300SW SEC 7.5 × 30  mm column and HLB Oasis cartridge were purchased from Waters (MA, USA), and Amicon Ultra-15 Centrifugal Filter Units from Millipore (Billerica, MA). *E. coli* BL21(DE3) cells were acquired from Novagen (Madison, WI), lambda integrase and excisionase from Elpis Biotech (Deajon, Korea), and dialysis membranes from Viskase (Darien, IL). Amicon Ultra was purchased from Merck Millipore (Darmstadt, Germany), Triton X-114 and 3-(4,5-dimethylthiazol-2-yl)−2,5-diphenyltetrazolium (MTT) from Sigma-Aldrich (St. Louis, MO), and polyethylenimine ‘Max’ (PEI) from Polysciences (Warrington, PA). The β-klotho plasmid was a gift from Dr. Zipora Yablonka-Reuveni (Addgene plasmid #45531). Commercial hFGF21 purified from inclusion bodies in *E. coli* (Cat# CYT-474) was acquired from Prospec (East Brunswick, NJ), and Dulbecco’s Modified Eagle’s Medium, foetal bovine serum, and penicillin-streptomycin from Gibco (MA).

### Construction of plasmids encoding hFGF21

For construction of hFGF21-expression vectors, the Gateway cloning system based on BP and LR recombination reactions was used^17^. A DNA codon-optimised sequence encoding 181 amino acid residues of mature hFGF21 (GenBank: AAQ89444.1) was synthesised (GenScript, Piscataway, NJ). The tobacco etch virus protease recognition site (TEVrs), ENLYFQ/G, was placed at the N terminus of the hFGF21 polynucleotide. The synthesised DNA sequence was then subcloned into pDONR207 via a BP reaction to obtain an entry clone named pENTR-hFGF21. Following the BP reaction, expression vectors were created by means of LR reactions between the pENTR-hFGF21 vector and one of eight destination vectors: pDEST-HGWA, pDEST-SUMO, pDEST-HXGWA, pDEST-HGGWA, pDEST-HMGWA, pDEST-HNGWA, pDEST-PDI, or pDEST-PDIb′a′. The correct clones of all the expression constructs were confirmed by sequencing (Macrogen, Deajeon, Korea).

### Expression and solubility testing of fusion proteins in *E. coli*

The expression plasmids were transformed into *E. coli* BL21(DE3) cells, and a single colony of each clone was picked and inoculated into 5 mL of the Luria-Bertani (LB) medium containing 50 μg/mL ampicillin at 37°C overnight. The overnight-cultured cells were then transferred into a fresh LB medium containing ampicillin at a 1:100 ratio, and the cells were continuously cultured at 37°C and 200 rpm in a shaking incubator. To induce expression of the hFGF21 fusion proteins, 0.5 mM IPTG was added into the culture batch at OD_600_ of 0.6. At this step, the temperature was kept constant at 37°C for 5 h or decreased to 18°C with incubation for 18 h. Finally, the cells were harvested and analysed by SDS-PAGE using a 10% tricine gel to evaluate the expression levels.

### Purification of hFGF21 from *E. coli* and tag cleavage

The cultured cells were harvested from a 500-mL cultivation batch by centrifugation at 3,800 × *g* for 20 min at 4°C. The medium was removed, and the pellets were kept at −80°C for later use. To prepare the supernatant fraction, the thawed cell pellets were resuspended in 50 mL of immobilised metal ion affinity chromatography (IMAC) buffer (20 mM Tris-HCl, 500 mM NaCl, 5% glycerol [v/v], pH 8.0) and sonicated by means of an ultrasonic cell disruptor JY99-IIDN (Ningbo Scientz Biotechnology, Guangdong, China) until the cells were completely homogenised. The sonicated sample was centrifuged at 23,000 × *g* for 20 min at 4°C, and then the supernatant was collected and filtered through a 0.4-μm-pore membrane for further purification.

After equilibration with 5 column volumes (CVs) of IMAC buffer, a 5-mL HisTrap FF column was used to purify MBP-hFGF21 from the prepared supernatant. Non-specifically bound proteins were washed out from the column with 5 CVs of IMAC buffer containing 50 mM imidazole. The MBP-hFGF21 fusion protein was completely eluted from the column with 5 CVs of IMAC buffer containing 1 M imidazole. To prevent an inhibitory effect of NaCl and imidazole on TEV protease activity, the purified fusion protein was dialysed against NaCl-free IMAC buffer (20 mM Tris-HCl, 5% glycerol [v/v], pH 8.0) before digestion with TEV protease. After that, the fusion protein was incubated with TEV protease at a ratio of 1:10 (w/w) for 5 h at room temperature. For purification of the protein after tag removal, NaCl was added to the digested sample to a final concentration of 500 mM and loaded onto a 5-mL HisTrap FF column pre-equilibrated with 5 CVs of IMAC buffer. To elute the hFGF21 protein, the column was washed with 5 CVs of IMAC buffer containing 50 mM imidazole. The column was finally recovered by washing with 5 CVs of IMAC buffer containing 1 M imidazole to completely remove the MBP tags and TEV protease. Endotoxin was then removed, and pure hFGF21 was dialysed against PBS at 4°C and lyophilised for further experiments. The protein concentrations were determined by the Bradford method with BSA as a standard.

### Purification of TEV protease from *E. coli*

Plasmid pRK793 containing TEV protease sequence^18^ was transformed into *E. coli* BL21(DE3) competent cells for soluble expression. The cells were induced (when OD_600_ reached 0.6–0.8) with 0.5 mM IPTG at 30°C for 18 h. The supernatant was prepared by a method similar to the one for MBP-hFGF21 described above. Briefly, the protein solution was loaded onto a 20-mL HisTrap FF 16/10 column, which was equilibrated with 5 CVs of IMAC buffer. Five CVs of IMAC buffer containing 100 mM imidazole was used to remove non-specifically bound proteins from the column. TEV protease was eluted with 5 CVs of IMAC buffer containing 1 M imidazole, and immediately, 5 mM dithiothreitol (DTT) was added into the sample to prevent aggregation. The eluted sample was concentrated to 10–15 mL using Amicon Ultra-15 Centrifugal Filter Units. Then, it was loaded onto a HiPrep 16/10 Desalting column to remove NaCl completely, and the buffer was changed to stock buffer (50 mM Tris-HCl, pH 8.0, 1 mM EDTA, 0.1% Triton X-100 [v/v], 10% glycerol [v/v]). Next, 2.5 mM DTT was added to the sample, and TEV protease was stored at −80°C. The activity of TEV protease was approximately 2.0 U/μL (1 U of TEV protease cleaves ~90% of 5.5 μg of the fusion protein at 25°C).

### Electrophoresis and quantification of protein expression and solubility

Protein fractions were mixed with 5× sample buffer (312.5 mM Tris-HCl, pH 6.8, 50% glycerol, 5% SDS, 0.05% bromophenol blue, 100 mM DTT) before they were loaded onto a 10% tricine SDS-PAGE gel. The protein bands were stained with a Coomassie brilliant blue R-250 solution. The expression level, solubility, and purity of proteins were measured by means of ImageJ software (http://imagej.nih.gov/ij) and also quantified as in other studies^19–23^.

### Endotoxin removal

For this purpose, the sample was incubated with 1% Triton X-114 at 4°C for 30 min. Then, the sample-Triton X-114 mixture was left at room temperature for 10 min. The aggregates were removed by centrifugation at 23,000 × *g* for 10 min at room temperature^24^. Finally, the Endpoint Chromogenic Limulus Amebocyte Lysate test (Lonza, Basel, Switzerland) was conducted to evaluate the endotoxin concentration in the final product. Limulus Amebocyte Lysate was incubated with Triton X-114–treated hFGF21 at 37°C for 10 min before the substrate was added. Next, a stop agent (25% v/v glacial acetic acid) was added to the mixture, and the release of p-nitroaniline was evaluated by photometric measurement at 405–410 nm.

### Analysis of purified hFGF21 by high-performance liquid chromatography (HPLC)

To determine hFGF21 purity, the final product was analysed by size exclusion chromatography with HPLC (SEC-HPLC). A protein-pak 300SW SEC 7.5 × 300 mm column was equilibrated with more than 10 CVs of PBS, pH 7.4. The protein was loaded onto the column at a flow rate of 1 mL/min for 40 min. The elution peaks were detected at 280 nm, and the experiment was carried out at room temperature.

### Protein extraction and digestion

Aliquots of 100 µg of protein were reduced, alkylated, and digested according to the protocol below. Briefly, 60 µL of 9 M urea and 30 mM DTT in 10 mL of 100 mM Tris base (pH 8.0) were added to each sample and incubated for 30 min at 37°C. Each sample was allowed to cool at room temperature before 9 µL of 500 mM iodoacetamide (0.0925 g/mL) was added. The solution was incubated for 20 min at room temperature. To dilute the urea from 6.0 to 0.6 M, 771 µL of 100 mM Tris-HCl buffer (pH 8.0) was added to each sample. Then, each sample was digested with trypsin, Glu-C, ASP-N, and chymotrypsin at a protein-to-enzyme ratio of 50:1 at 37°C overnight. The digestion was quenched with 50 µL of 0.1% formic acid. After that, the digested peptide mixture was applied to an HLB Oasis cartridge for desalting, and peptides were eluted with 1 mL of 40% and 60% acetonitrile in a 0.1% formic acid solution.

### Protein identification by Q-Exactive analysis

For identification of the hFGF21 proteome, nano-liquid chromatography with tandem mass spectrometry (nano-LC-MS/MS) experiments were performed on an NanoAcquity ultra-performance liquid chromatography (UPLC) system that was connected to a Q-Exactive spectrometer (Thermo Scientific, Bremen, Germany) through a nanoelectrospray ion source. Peptides were loaded by an autosampler onto a pre-column (2 cm long, internal diameter [ID] 180 μm, particle size 5 μm) and analytical column (10 cm long, ID 150 μm, particle size 1.7 μm) that was packed with reversed-phase C18 resin. In addition, the samples were loaded at a flow rate of 300 nL/min. The peptides were separated with a linear acetonitrile gradient from 3% to 40% for 30 min and from 40% to 60% for 20 min.

The Q-Exactive instrument was operated in data-dependent mode (DDA) to switch automatically between full-scan MS and MS/MS modes of acquisition. A survey of full-scan MS spectra (350−1600 m/z) was conducted on the Orbitrap at resolution 70,000 at 200 m/z after ions accumulated to a target value of 3 × 10^6^, based on predictive AGC from a previous full scan. The dynamic exclusion was set to 30 s. The 12 most intense multiply charged ions (z ≥ 2) were sequentially isolated and fragmented by higher-energy collisional dissociation (HCD) in an octopole collision cell with fixed injection time 60 ms, an AGC target value of 5 × 10^4^, and resolution of 17,500. The typical mass spectrometric conditions were as follows: S-lens RF level, 65; spray voltage, 2 kV; heated capillary temperature, 320°C; and normalised HCD collision energy, 30%. The under-fill ratio, MS/MS ion selection threshold, and isolation width were set to 1%, 8.3 × 10^3^ counts, and 2 m/z, respectively. The fixed first m/z was set to 100.

### Database searches

These searches (SEQUEST) were conducted using Proteome Discoverer (Thermo Fischer Scientific, ver. 1.4.0.288). The MS/MS data were queried against hFGF21 using the following parameters: MS accuracy, 10 ppm; MS/MS accuracy, 0.8 Da for HCD; trypsin digestion with 2 missed cleavages allowed; fixed carbamidomethyl modification of cysteine, +57.0215 Da; and variable modification of oxidised methionine, +15.9949 Da.

### Disulphide bond identification by MALDI-TOF/TOF MS

The purified hFGF21 (20 µg) was alkylated with 10 mM iodoacetamide (IAA) for 20 min at room temperature in the dark. Trypsin (1.4 µg) was then added to the sample and the reaction was left at 37°C overnight to yield non-reduced sample. For the preparation of reduced sample, the protein was incubated with 10 mM DTT for 10 min at 95°C prior to alkylation and trypsinisation. All the samples were desalted, concentrated by Ziptip C18 (Millipore, Billerica, MA) and then mixed with matrix α-Cyano-4-hydroxycinnamic acid. The analysis was conducted using MALDI-TOF/TOF™ 5800 (AB SCIEX, Framingham, MA) at the Korea Basic Science Institute (Seoul, Korea).

### Cell culture and transient transfection

Transfection of the β-klotho gene was carried out in NIH-3T3 cells kindly provided by Dr. David Kaplan. The cells were cultured in Dulbecco’s Modified Eagle’s Medium supplemented with 10% of foetal bovine serum and 1% penicillin-streptomycin at 37°C and 5% CO_2_ with saturating humidity. One day before the transfection, approximately 2 $\times$ 10^5^ cells were seeded in a 48-well plate containing 500 µL of the complete medium per well. The medium was then replaced by 400 µL of a fresh medium without penicillin-streptomycin, and the cells were transfected with the β-klotho plasmid using PEI. Namely, 0.5 µg of the plasmid and 1.5 µg of PEI were mixed and incubated for 30 min at room temperature. Into each well containing cells to be transfected, the plasmid–PEI mixture was added to the final volume of 500 µL, and then the plate with transfected cells was incubated for 24 h.

### An hFGF21 bioactivity assay

The activity of hFGF21 was assessed using β-klotho–transfected NIH-3T3 cells. After 24 h of transfection, the old medium was removed, and the transfected cells were grown in a starvation medium containing 1% of foetal bovine serum. To determine the protein’s bioactivity, the transfected NIH-3T3 cells in 48-well plate were incubated directly with different concentrations of purified hFGF21 or commercial hFGF21 (0.01, 0.1, 1, 5, 10, 50, 100, 250, or 500 ng/mL). The cell viability was calculated by means of the MTT assay after 24 h of incubation with the proteins. All the protein concentrations were tested in triplicate.

### Data analysis

The data was analyzed by GraphpadPrism 5 software (GraphPad, San Diego, CA) and P $\le$ 0.05 was significant. The data was processed using following equation and Microsoft Excel software:

1 $$\begin{array}{c}Re=Bl+(Max\mbox{--}Bl)/(1+{(E{C}_{50}/conc)}^{Hs})\\ \quad \quad -(Max\mbox{--}Bh)/(1+{(I{C}_{50}/conc)}^{Hi})\end{array}$$

where *Re* is response of cells, *Max* is maximum response, *conc* is a concentration of the protein, *Bh* is baseline at high concentration, and *Hs, Hi* is Hill coefficient of stimulation and inhibition.

All data are presented as a mean $\pm$ standard error (SE) of n $\ge$ 3 independent experiments. The statistical significance of the responses was performed by two-tailed Student’s t-test using Graphpad Prism 5 software (GraphPad, San Diego, CA) and the significance was considered as p < 0.05.

## Results

### Construction of eight expression vectors of hFGF21

**Figure 1:**
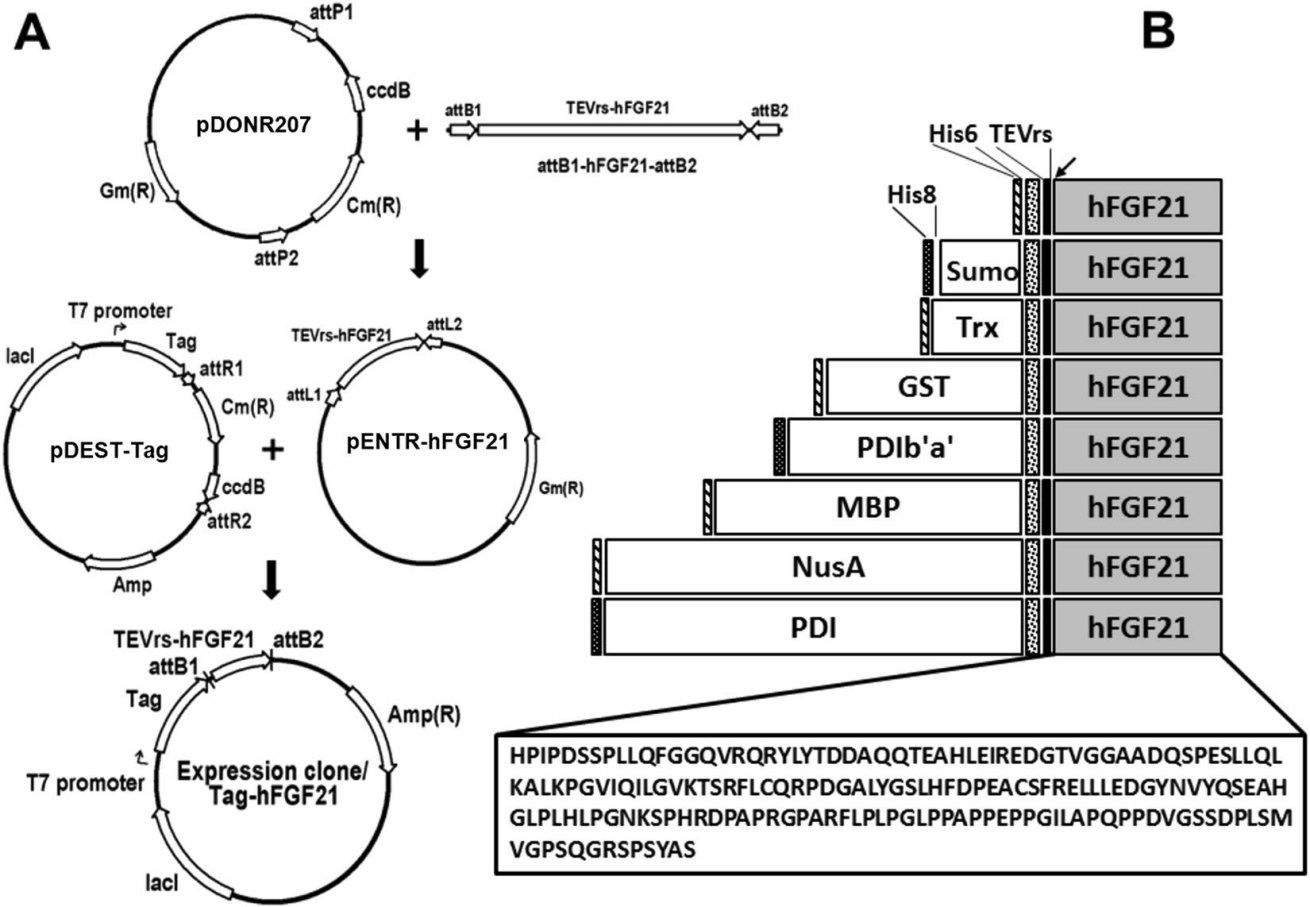
Construction of the hFGF21 expression vectors and schematic representation of the domain structure of fusion proteins. (**A**) The vector map of pHMGWA-hFGF21 for the Gateway® cloning method. Gene expression of the fusion proteins is under control of the IPTG-inducible T7 promoter. Ampicillin served as a selection marker. (**B**) Schematic structure of the eight tagged proteins His6-, Sumo-, Trx-, GST-, PDIb′a′-, MBP-, NusA-, and PDI-hFGF21 (total size). The arrow indicates the TEV protease cleavage site between the tags and hFGF21.

To enhance the expression and solubility of hFGF21 in the cytoplasm of *E. coli*, hFGF21 was fused with each of eight tags – His6, Sumo, Trx, GST, PDIb′a′, MBP, PDI, and NusA – at the N terminus, creating the expression plasmids shown in Fig. 1. A TEVrs was inserted between the tags to allow for separation during the purification process. To utilise the highly efficient nickel NTA chromatography system, we also included a His6 sequence at the N terminus of Trx, GST, MBP, NusA, and a His8 sequence at the N terminus of Sumo, PDI, and PDIb′a′ (Fig. 1B). These codon-optimised plasmids were transformed into the *E. coli* BL21(DE3) strain to test the expression and solubility caused by the tag candidates.

### Prokaryotic expression of tagged hFGF21 proteins in *E. coli*

**Figure 2:**
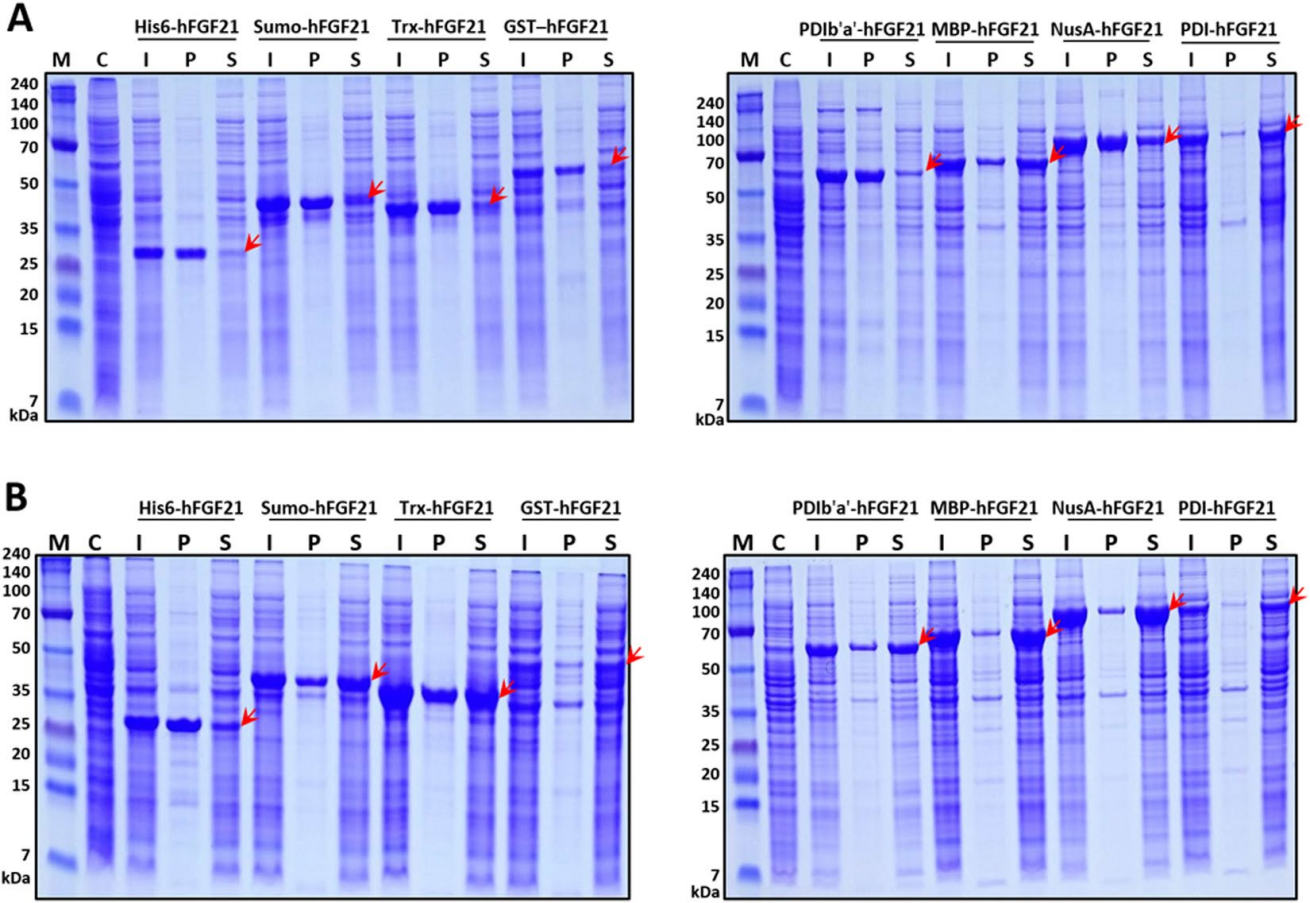
Expression levels of eight hFGF21 fusion proteins in *E. coli* BL21(DE3). Expression of the fusion proteins was induced by the addition of 0.5 mM IPTG at 37°C (**A**) or 18°C (**B**). The arrows indicate migration of hFGF21 fused with each tag in a polyacrylamide gel. M, molecular weight markers; C, total cell protein before IPTG induction as a negative control; I, whole cells after IPTG induction; P, the pellet fraction after cell sonication; S, the supernatant after cell sonication.

Expression of the hFGF21 fusion variants was mediated by the T7 promoter, and production was induced by 0.5 mM IPTG addition under two different temperature conditions: 37°C and 18°C. The expression levels of tagged hFGF21 proteins varied at these parameters, ranging from 10% to 43% (Fig. 2 and Table 1). In general, the expression of hFGF21 fusion proteins at 18°C was higher than that at 37°C (Fig. 2). The expression of the PDIb′a′ construct was equivalent at the two temperatures. Lowering the temperature decreased the expression levels of GST-tagged hFGF21 from 17.8% to 10.6%. The solubility of the proteins was also temperature and tag dependent. PDI-hFGF21 and MBP-hFGF21 differed from the other fusion variants in the solubility of the resulting proteins. At 37°C, where most of the fusion proteins formed inclusion bodies, more than 60% of the MBP- and PDI-fused proteins were expressed in soluble form (Fig. 2A and Table 1). Lowering the induction temperature to 18°C markedly enhanced the solubility of most of the candidate proteins (Fig. 2B and Table 1). Also shown in Table 1 are the solubility levels of each fusion protein that were greatly increased to more than 85% using the PDIb′a′, MBP, PDI, and NusA tags. Because of the high expression and solubility and small size of the MBP tag, it was chosen for purification optimisation and downstream functionality testing.

**Table 1:** Expression and solubility levels of hFGF21 fused with eight different tags.

Tag	Tag size (kDa)	Fusion protein size (kDa)	Expression level (%)		Solubility (%)	
			**37** ^**o**^ **C**	**18** ^**o**^ **C**	**37** ^**o**^ **C**	**18** ^**o**^ **C**
His6	0.8	24.1	25.8	29.1	11.5	36.2
Sumo	11.3	35.8	29.4	31.8	48.8	63.5
Trx	11.7	35.9	29.2	36.4	12.4	48.0
GST	25.6	49.8	17.8	10.6	15.6	62.2
PDIb′a′	30.6	55.1	29.7	29.8	23.1	85.1
MBP	40.2	64.4	35.9	40.6	60.5	88.6
NusA	54.7	79.0	37.7	43.5	23.2	89.1
PDI	54.8	79.3	13.6	15.1	82.1	88.3

### Purification of hFGF21 from the MBP-hFGF21 fusion protein

**Figure 3:**
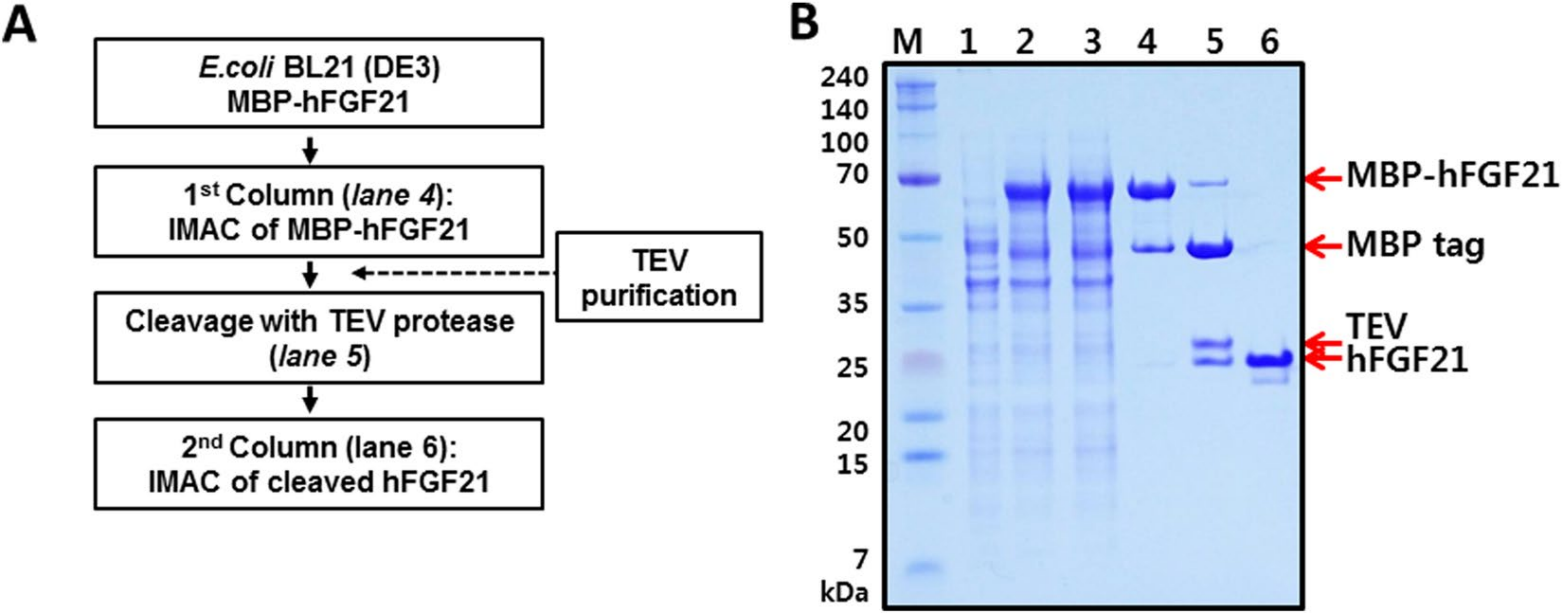
Purification of hFGF21 from soluble MBP-hFGF21 expressed in *E. coli* BL21(DE3). (**A**) The flowchart of the purification process. (**B**) The fusion protein and hFGF21 after tag cleavage were both purified by IMAC. M, molecular weight makers; lane 1, total cell protein before IPTG induction as a negative control; lane 2, whole-cell lysate after IPTG induction; lane 3, the soluble fraction after cell sonication; lane 4, the MBP-hFGF21 fusion protein purified by IMAC (64.4 kDa); lane 5, the MBP tag was cleaved by TEV protease: the MBP tag (40.3 kDa), hFGF21 (19.45 kDa); lane 6, the final hFGF21 product (19.45 kDa).

hFGF21 from the MBP-hFGF21 variant was purified by two rounds of immobilised metal ion affinity chromatography (IMAC). The sequential purification is important because the first IMAC is intended to obtain the fusion protein, and the subsequent IMAC is needed to separate the target protein from the tag after TEV protease treatment (Fig. 3A). BL21(DE3) *E. coli* transformed with the MBP-hFGF21 expression vector was induced with 0.5 mM IPTG at 18°C for 18 h and then harvested. In comparison with cells not exposed to IPTG, the induced cell fraction showed the desired protein band representing recombinant MBP-hFGF21 (Fig. 3B, lanes 1 and 2). The cells were disrupted by sonication, and the supernatant was collected by centrifugation. No significant difference between whole-cell and supernatant fractions was observed, indicating that most of the fusion proteins remained soluble after sonication (Fig. 3B, lane 3). The filtered supernatant was then loaded onto nickel chromatography columns, and MBP-hFGF21 was eluted after a washing step to remove impurities from the column. The SDS-PAGE results showed that the eluted sample contained the MBP-hFGF21 protein at purity ~73.1% (Fig. 3B, lane 4, and Table 2).

**Table 2:** Prokaryotic purification of hFGF21 from solubly expressed MBP-FGF21.

Purification step	Total protein (mg)	Purity (%)	hFGF21 (mg)	Yield (%)
Cell weight	1900 (pellet)	—		—
Supernatant	252.0	40.6	30.9	100
1^st^ IMAC	50.0	73.1	11.0	34.0
2^nd^ IMAC	8.5	95.4	8.1	26.2

**Figure 4:**
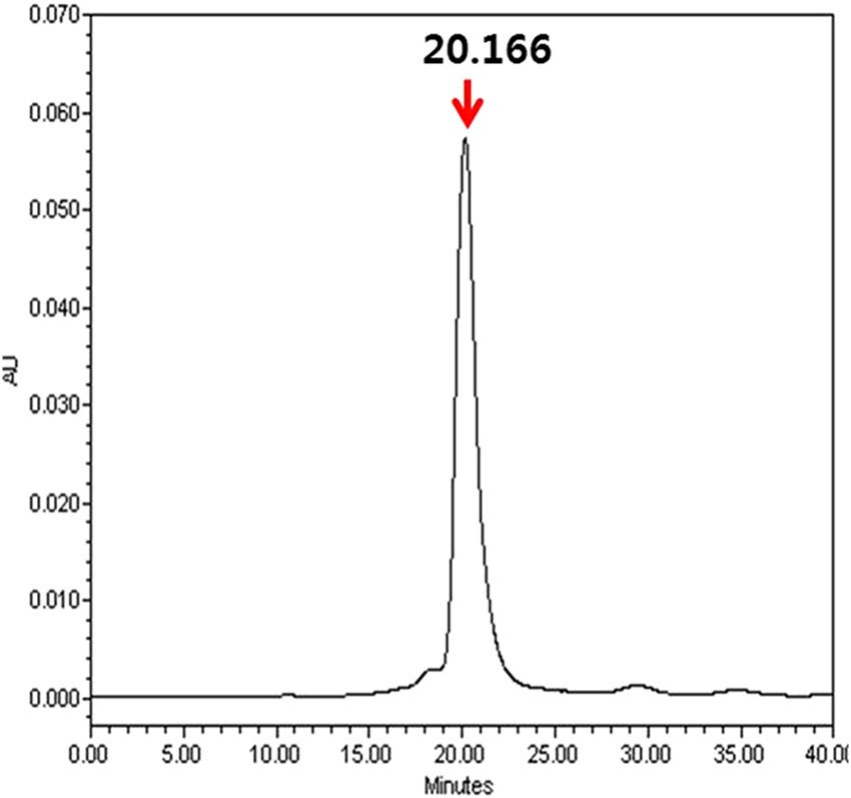
SEC-HPLC analysis of hFGF21. The purified hFGF21 was analysed by HPLC using a protein-pak 300SW SEC 7.5 × 300 mm column to evaluate the purity. The x-axis indicates retention time (min), while the y-axis shows the absorbance at 280 nm (arbitrary units, AU). The main peak of pure hFGF21 appeared at 20.166 min.

This purified MBP-hFGF21 protein was then subjected to TEV protease cleavage. As shown in Fig. 3B, lane 5, incubation at room temperature for 5 h resulted in highly efficient MBP-hFGF21 cleavage. Next, a HisTrap FF column was used to separate the hFGF21 from the tag removal reaction mixture. Cleaved hFGF21 effectively bound to the column and was readily eluted with 50 mM imidazole-containing buffer; the purity of the peptide was 95.4% as shown in Fig. 3B, lane 6, and Table 2. The MBP tag, undigested MBP-hFGF21, and TEV protease bound tightly to the column owing to the His6 tag present at their N terminus and were removed by washing the column with the buffer containing 1 M imidazole. Under these experimental conditions, a total of 8.1 mg of tag-free hFGF21 was obtained from 500 mL of starting cell culture (Table 2). To determine the purity of the final hFGF21 product, SEC-HPLC was performed, and the major peak of hFGF21 appeared at 20.166 min. The chromatogram revealed that the purity of hFGF21 was approximately 96% (Fig. 4). After that, the target protein was treated with Triton X-114, and the endotoxin level was estimated. The endotoxin concentration in our purified hFGF21 was lower than 0.1 EU/μg, which is in the range of commercially and clinically available recombinant peptides.

### Characterisation of the FGF21 protein

**Figure 5:**
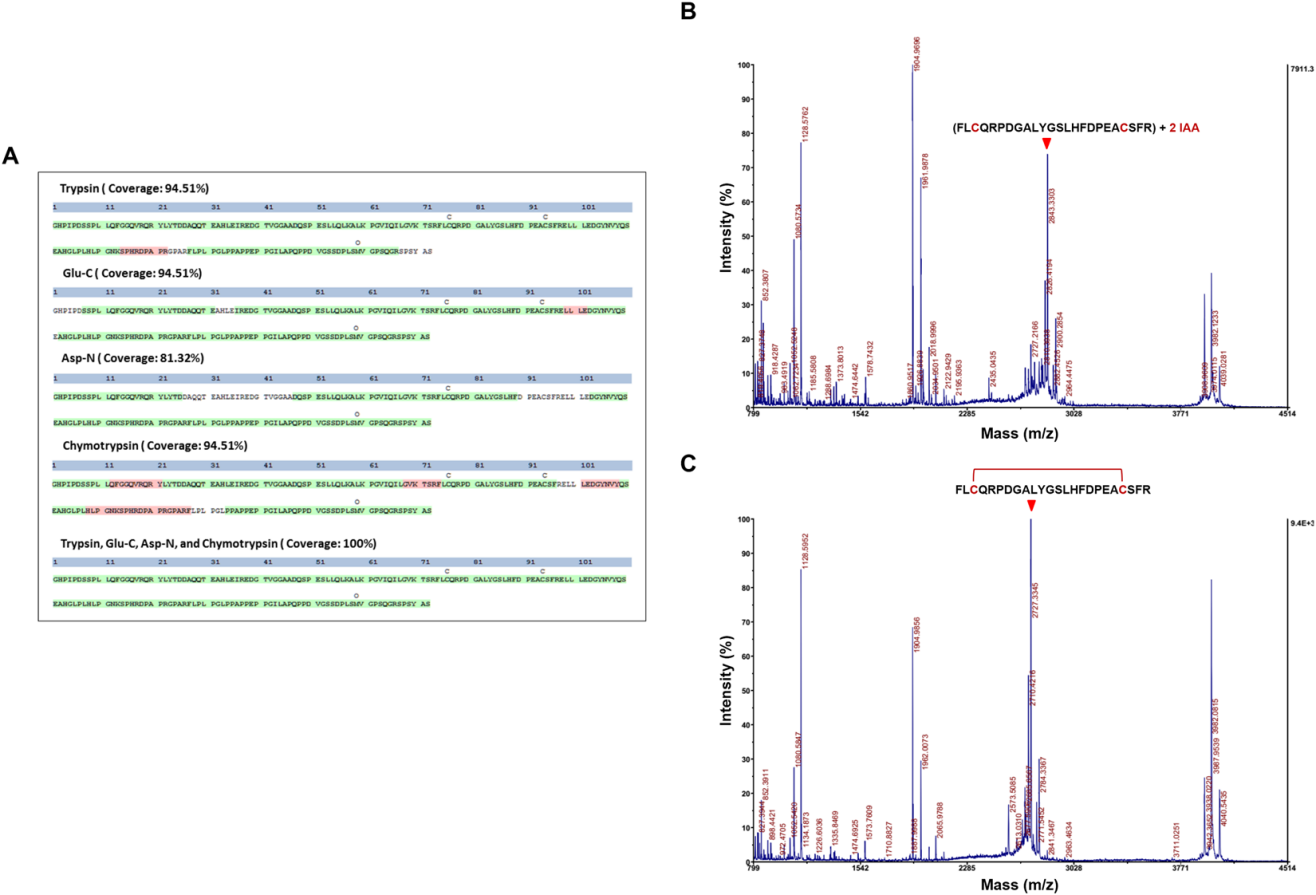
Protein and disulphide bond identification using mass spectrometry-based analysis. (**A**) LC-MS/MS sequencing of peptides identifying the proteins was performed on samples digested with four enzymes (trypsin, Glu-C, ASP-N, and chymotrypsin). Percent coverage for protein identification is represented by the identified peptides in the total protein sequence; trypsin: 94.51%, Glu-C: 94.51%, Asp-N: 81.32%, chymotrypsin: 94.51%, and all four enzymes: 100%. Each sequence confidence is represented by PMSes as illustrated by high (green) and low (red) Percolator confidence scores. MALDI-TOF/TOF MS for purified hFGF21 in reducing condition (**B**) and non-reducing condition (**C**). The arrow indicates the peptide fragment with either the two IAA-alkylated cysteines or the formed disulphide bond.

To confirm that the full-length intact hFGF21 protein was produced, we performed enzymatic digests with trypsin, Glu-C, ASP-N, and chymotrypsin; LC-MS/MS analysis was then conducted. Using the multi-consensus identification approach, hFGF21 was confirmed with 100% sequence coverage (Fig. 5A). The MS/MS spectra of the identified peptides in hFGF21 are summarised in Supplementary Table 1.

To determine the presence of a disulphide bond between Cys75 and Cys93, the purified hFGF21 was trypsinised and alkylated by iodoacetamide (IAA) with or without DTT, and the mass analysis was performed using MALDI-TOF/TOF MS. A spectral peak with m/z = 2843.3303 was observed, indicating that the two cysteines were IAA-alkylated under reducing condition (Fig. 5B). However, under non-reducing condition, a reduction in the m/z value to 2727.3345 indicated that the two cysteines were not alkylated by IAA (Fig. 5C). These data point the presence of a covalent disulphide bridge between Cys75 and Cys93 in the purified hFGF21.

### Bioactivity

**Figure 6:**
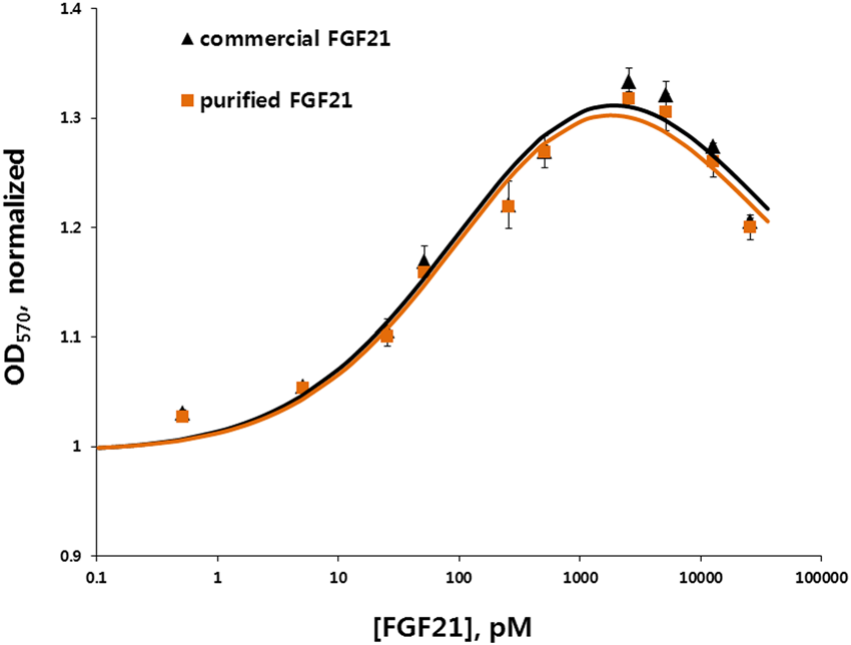
A bioactivity assay of purified hFGF21 in a transfected NIH-3T3 cell line. The effect of the purified hFGF21 was determined in NIH-3T3 cells transfected with β-klotho. Dose-response proliferation curves of the cells were constructed from various concentrations of commercial hFGF21 from *E. coli* source and purified hFGF21.

Previously, it has been shown that β-klotho is activated after FGF receptor engagement by hFGF21^25,26^. Hence, the β-klotho gene was transfected into and expressed in the NIH-3T3 cell line. The effects of recombinant hFGF21 on transiently transfected NIH-3T3 cells was determined by a cell proliferation assay. Various concentrations of purified and commercial hFGF21 were tested. After 24 h of incubation, the MTT assay was used to measure proliferation of the cells. The results showed that the β-klotho–transfected NIH-3T3 cells were stimulated with either purified or commercial hFGF21 in a dose-dependent manner. At concentrations less than or equal to 5 nM, the dose-response curves had a sigmoidal pattern. In contrast, higher concentrations showed a decreasing proliferation response of the cells, resulting in a bell-shaped curve (Fig. 6). The half maximal effective concentrations (EC_50_s) of commercial and purified hFGF21 were 300.01 ± 0.00 and 300.03 ± 0.01 pM, with Hill coefficients of 0.48 ± 0.01 and 0.50 ± 0.02, respectively. The differences in EC_50_s and the Hill coefficients were statistically insignificant. Untransfected cells showed no proliferative effects for both purified and commercial hFGF21s (data not shown). These data indicate the ability of our engineering and purification process to robustly generate and isolate hFGF21 in a manner that is indistinguishable from commercial sources of the recombinant polypeptide.

## Discussion

The main purpose of this study was to determine and optimise the conditions for overexpression and solubilisation of hFGF21 in the cytoplasm of *E. coli* to facilitate purification of the candidate protein. To this end, one of eight tags – His6, Sumo, Trx, GST, PDIb′a′, MBP, NusA, or PDI – was attached to the N terminus of hFGF21, resulting in eight recombinant vectors. Previously, these tags have been used in multiple studies to improve the soluble expression of various recombinant proteins in *E. coli* ^19–23,27,28^. In the present study, the hFGF21 fusion vectors were transformed into the *E. coli* BL21(DE3) to characterise the behaviour of the fusion proteins expressed in the cytoplasm. At two expression temperatures, 37°C and 18°C, the expression of all the constructs was relatively high, with various solubility levels among the tagged proteins (Table 1). At 37°C, only the MBP and PDI tags could enhance the solubility to greater than 60%, whereas the other fusion proteins were primarily expressed as inclusion bodies. It has been suggested that high induction temperature causes protein aggregation into inclusion bodies, whereas lowering of the temperature allows for soluble expression and folding of the proteins expressed in *E. coli* ^29^. In agreement with these observations, lowering the temperature to 18°C elicited solubility enhancement of all the expressed constructs, up to more than 80% in the case of PDIb′a′, MBP, NusA, and PDI tags. These results indicate that a low temperature for hFGF21 protein induction is a beneficial factor for expression and correct folding in *E. coli*.

Because of its small size and high expression and solubility, MBP-hFGF21 was selected for further purification and characterisation. Data from many studies have shown multiple uses of MBP as a fusion partner to prevent inclusion body formation and to promote soluble expression and purification of proteins of interest^19–22,27^. Nonetheless, the downside of MBP as a tag is that once this tag is removed, the target proteins tend to become unstable and form aggregates. Furthermore, to purify a protein of interest from a mixture, rigorous washing steps are necessary to completely remove free maltose in order to prevent it from rebinding to an amylose column^30^. To address these problems here, a His6 tag was attached to the N terminus of the MBP tag to provide additional flexibility in the purification of the recombinant proteins by IMAC^31^. Indeed, the His6-MBP combination not only facilitated the protein expression in soluble form but also allowed for straightforward downstream purification. This notion is supported by the IMAC purification rate of 73%. After the TEV enzyme digestion, the other proteins such as free MBP tag, TEV protease, and a small amount of undigested MBP-hFGF21 were readily removed by reloading the cleaved mixture onto the second IMAC. Overall, 8.1 mg of soluble hFGF21 was obtained with 96% purity by means of these two simple purification steps (Table 2). Recently, hFGF21 was purified from the cytoplasm of *E. coli* using the Sumo tag and affinity chromatography^10^. However, the soluble expression of Sumo-hFGF21 was significantly lower than that of MBP-hFGF21 as shown in Fig. 2. In addition, our purification of hFGF21 requires only two chromatography steps taking approximately 15 h. This short handling time and the simple protocol could reduce the degradation and increase the yield and bioactivity of the target protein. Endotoxins which are highly toxic and immunogenic to humans can be co-purified with recombinant proteins produced from most of *E. coli* hosts, hampering their use in biological and clinical research^32,33^. Therefore, the endotoxin threshold for a clinical use of recombinant proteins from *E. coli* is set to be less than 1 EU/μg^34^. In our study, the endotoxin level of the final purified hFGF21 was lower than 0.1 EU/μg, thus satisfying the safety standard.

FGF proteins commonly require heparan sulphate proteoglycan for the interaction with their receptors (FGFRs)^35^. Exceptionally, FGF21 lacks a heparin-binding domain^2,36^ and instead requires β-klotho as an obligate coreceptor^25,37,38^. Several studies have shown that the C-terminus of the protein is responsible for the interaction to β-klotho whereas its N-terminus interacts to FGFRs^39,40^. After the tag cleavage by TEV protease, one glycine residue was remained at the N-terminus of the purified hFGF21. However, in our cell proliferation assay using NIH-3T3 cells transfected with a β-klotho gene, we observed that the ED50s of the purified hFGF21 and also the commercial protein were about 300 pM (Fig. 6). This result shows that the extra glycine residue at the N-terminus of the protein does not affect the biological activity and our *E. coli*-derived hFGF21 shows proper folding and interaction with β-klotho and its receptors in a live-cell setting. Our result is also in good agreement with previous studies that a modification at the N-terminus of hFGF21 by a fusion with Fc or deletion of the first four amino acids does not affect the activity^16,41^.

Due to lack of protein glycosylation machinery, the hFGF21 produced in *E. coli* is non-glycosylated. Serine at position 167 is the major site for *O*-linked glycosylation of hFGF21. However, the substitution of serine to alanine resulting in devoid of glycosylation does not appear to negatively impact to the hFGF21 functionality^16^. In addition, many studies on the biological function or receptor interaction and activation have been done using non-glycosylated hFGF21 purified from *E. coli* ^3,5,13,25,40^, suggesting that the glycosylation may be dispensable for the functional activity and receptor binding of hFGF21.

Based on the elucidated structure of the other FGF members, the FGF21 structure was modelled to have an intramolecular disulphide bond between the two cysteine residues, Cys75 and Cys93^16,42,43^. Our mass spectrometry results demonstrated experimentally for the first time that indeed the disulphide bond was formed. As shown in Fig. 5C, in non-reducing conditions, the peak alkylated with IAA disappeared whereas only the new peak corresponding to the exact size of disulphide-bonded peptide appeared, demonstrating that the disulphide bond presents in most of the final hFGF21 product under our engineering conditions. The reducing environment of *E. coli* cytoplasm does not favour the formation of disulphide bonds of recombinant proteins, resulting in protein miss-folding and aggregation^44^. Our results suggest that, by fusion to MBP tag which functions as a “holdase” to stabilise incompletely folded protein^45,46^, hFGF21 may be properly folded to its native conformation, hence, increasing the rates of disulfide bond formation even in the cytoplasm or upon exposure to oxidizing conditions after lysis of the *E. coli* cells. This disulphide bond is thought to be conserved across several subfamilies and homologues of the FGFs and stabilize two of the FGF β-hairpins, enhancing the correct folding and stability of the proteins^43^.

hFGF21 is a highly promising drug candidate owing to its therapeutic effects on metabolic diseases^5,7,8^. Our study describes a robust method for the expression and purification of recombinant hFGF21 in soluble form in the cytoplasm of *E. coli*. The construction of hFGF21 fused with different tags was designed to compare their expression and solubility in *E. coli*. The MBP fusion protein was then chosen for the purification of hFGF21. By means of only a single conventional chromatography technique, 8.1 mg of bioactive hFGF21 with high purity was obtained from 1.9 g of wet weight in00 mL of cell culture. In conclusion, our strategy represents a highly efficient and cost-effective purification platform for prokaryotic production of bioactive hFGF21.

## Electronic supplementary material

Supplementary Dataset 1
